# Antioxidant Activities of Hot Water Extracts from Various Spices

**DOI:** 10.3390/ijms12064120

**Published:** 2011-06-21

**Authors:** Il-Suk Kim, Mi-Ra Yang, Ok-Hwan Lee, Suk-Nam Kang

**Affiliations:** 1 Department of Animal Resources Technology, Gyeongnam National University of Science and Technology, Gyeongnam, 660–758, Korea; E-Mails: iskim@gntech.ac.kr (I.-S.K.); karisto2000@nate.com (M.-R.Y.); 2 Department of Food Science and Biotechnology, Kangwon National University, Chuncheon, 200–701, Korea

**Keywords:** spices, antioxidant activity, radical scavenging activity, total phenolic content, total flavonoid content

## Abstract

Recently, the natural spices and herbs such as rosemary, oregano, and caraway have been used for the processing of meat products. This study investigates the antioxidant activity of 13 spices commonly used in meat processing plants. The hot water extracts were then used for evaluation of total phenolic content, total flavonoids content and antioxidant activities. Our results show that the hot water extract of oregano gave the highest extraction yield (41.33%) whereas mace (7.64%) gave the lowest. The DPPH radical scavenging ability of the spice extracts can be ranked against ascorbic acid in the order ascorbic acid > clove > thyme > rosemary > savory > oregano. The values for superoxide anion radical scavenging activities were in the order of marjoram > rosemary > oregano > cumin > savory > basil > thyme > fennel > coriander > ascorbic acid. When compared to ascorbic acid (48.72%), the hydroxyl radical scavenging activities of turmeric and mace were found to be higher (p < 0.001). Clove had the highest total phenolic content (108.28 μg catechin equivalent (CE)/g). The total flavonoid content of the spices varied from 324.08 μg quercetin equivalent (QE)/g for thyme to 3.38 μg QE/g for coriander. Our results indicate that hot water extract of several spices had a high antioxidant activity which is partly due to the phenolic and flavonoid compounds. This provides basic data, having implications for further development of processed food products.

## 1. Introduction

Oxidation is one of the major causes of chemical spoilage, resulting in rancidity and/or deterioration of the nutritional quality, color, flavor, texture and safety of foods [1,2]. It is well known that reactive oxygen species (ROS) formed *in vivo*, such as superoxide anion, hydroxyl radical and hydrogen peroxide, are highly reactive and potentially damaging transient chemical species. The oxidative damages caused by ROS on lipids, proteins and nucleic acids may trigger various chronic diseases, such as coronary heart disease, atherosclerosis, cancer and aging [3,4]. Antioxidant refers to a compound that can delay or inhibit the oxidation of lipids or other molecules by inhibiting the initiation or propagation of oxidative chain reactions and can thus prevent or repair the damage done to the body’s cells by oxygen [5].

Plants, including herbs and spices, have many phytochemicals which are a potential source of natural antioxidant, e.g., phenolic diterpenes, flavonoids, alkaloids, tannins and phenolic acids [6–8]. Natural antioxidants are known to protect cells from damage induced by oxidative stress, which is generally considered to be a cause of aging, degenerative diseases, and cancer [9]. These health promoting effects of antioxidants from plants and spices are thought to arise from their protective effects by counteracting ROS. Spices, like turmeric, fenugreek, mustard, ginger, *etc*. may offer many health benefits and have been proven to counteract oxidative stress *in vitro* and *in vivo* [10,11]. Most of these spices have been intensely studied only for their active components like phenolic acids and flavonoids [12,13].

Although the efficacy and mechanisms of action of spices have not been tested scientifically in most cases, these simple medicinal preparations often mediate beneficial responses due to their active chemical constituents [14]. Therefore, the objective of this study was to determine the total phenolic and total flavanoid contents and antioxidant properties of spices used in meat processing plants and to investigate the relationship between phenolic content and antioxidant activity.

## 2. Results and Discussion

### 2.1. Extract Yields, Total Phenolic Contents, and Total Flavonoid Contents

Table 1 lists the 13 spices used in this study, as well as their extraction yields, and total phenolic and total flavonoids contents. The differences between the extracts for these parameters were statistically significant (p < 0.001). The extraction yields of hot water extracts obtained for the 13 species ranged from 41.33% to 7.64%. The extraction yield can be ranked as oregano > thyme > basil > rosemary, clove, marjoram and savory > coriander > fennel, turmeric and caraway > cumin > mace. These results are in good agreement with the study by Hinneburg *et al.* [15], in which water extraction yields of several spices were ranged from 88 mg/g (8.8%) to 422 mg/g (42.2%).

The crude hot water extracts of the present study were used for comparison of their antioxidant activities, *i.e.*, DPPH radical scavenging activity, hydroxyl radical scavenging activity, and superoxide anion radical scavenging activity. The total phenolic and total flavonoids contents were also determined for the hot water extracts, using colorimetric methods.

Phenolic substances have been shown to be responsible for the antioxidant activity of plant materials [16]. The total amount of phenolic and flavonoids present in the water extracts of the selected spices are shown in Table 1. Clove had the highest total phenolic content (108.28 μg CE/g), while mace had the lowest value (6.50 μg CE/g), using the standard curve of catechin (R^2^ = 0.9482). The total phenolic content in decreasing order was clove > thyme > savory > rosemary > oregano, basil and marjoram > caraway, cumin, fennel, coriander, turmeric and mace (p < 0.001).

Flavonoid as one of the most diverse and widespread group of natural compounds are probably the most important natural phenolics. These compounds possess a broad spectrum of chemical and biological activities including radical scavenging properties [16,17]. Using the standard curve of quercetin (R^2^ = 0.9825), the total flavonoid content of spices varied from 324.08 μg QE/g for thyme to 3.38 μg QE/g for coriander. The total flavonoid content in decreasing order was thyme > rosemary > marjoram and oregano > basil > cumin > clove > caraway and fennel > savory > turmeric, mace and coriander (p < 0.001). It is known that the higher the phenolic and flavonoid contents of spices, the higher its antioxidant activities. Rice-Evans *et al.* [18] reported that the antioxidant properties of phenolic acids and flavonoids are due to their redox properties, ability to chelate metals and quenching of singlet oxygen.

### 2.2. DPPH Radical Scavenging Activity

**Figure 1:**
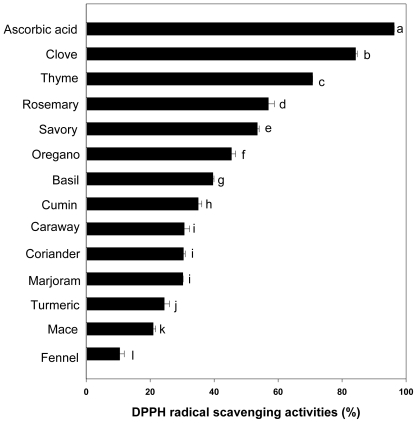
DPPH radical scavenging activities of hot water extracts of spices at a concentration of 1 mg/mL (n = 6, error bars represent standard deviation). ^a–l^ Values are significantly different between the samples (p < 0.001).

2,2-Diphenyl-1-picrylhydrazyl (DPPH) is widely used to test the ability of compounds to act as free radical scavengers or hydrogen donors, and to evaluate antioxidant activity of foods [1]. Figure 1 shows that the DPPH radical scavenging ability of the extracts can be ranked in the order clove (84.22%) > thyme (70.79%) > rosemary (56.98%) > savory (53.51%) > oregano (45.43%) > basil (39.63%) > cumin (35.02%) > caraway (30.67%), coriander (30.40%), marjoram (30.22%) > tumeric (24.43%) > mace (20.94%) > fennel (10.48%). The observed differential scavenging activities of the extracts against the DPPH system could be due to the presence of different compounds in the extract. Although the DPPH radical scavenging activities of the spices were less (p < 0.05) than those of ascorbic acid, the study revealed that most spices had free radical scavengers or inhibitors, acting possibly as primary antioxidants. In addition, a significant and linear relationship existed between the DPPH scavenging activity and phenolic content (Table 2), indicating that phenolic compounds are major contributors to antioxidant activity. The highly significant correlations obtained in this study support the hypothesis that phenolic compounds contribute significantly to the DPPH radical scavenging capacity of spice plants (r = 0.9158, p < 0.001). The good correlation between the results from total phenolics analysis and the antioxidative assays has been previously reported [19]. Moreover, Liu *et al.* [20] have reported the clove extract was significantly higher in the total phenol content and DPPH radical scavenging activity than other Chinese herbal plant. Although it is possible that the DPPH radical scavenging activity of spices could be mediated by individual phenolic acids, the overall antioxidant potential of spices are likely exhibited by the synergistic effect of the combinations of total phenolic acids and other antioxidant components including antioxidant vitamins considering the wide mixture of phenolic antioxidants present in spices extract.

### 2.3. Superoxide Anion Radical Scavenging Activity

**Figure 2:**
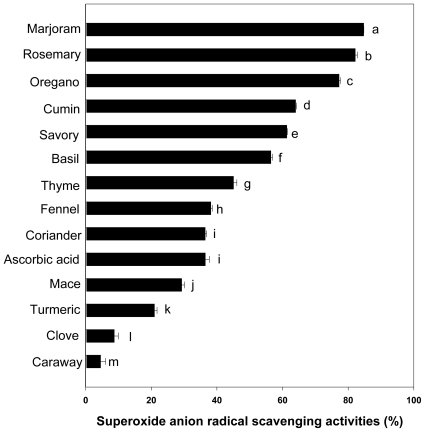
Superoxide radical scavenging activity of hot water extracts of spices at the concentration of 0.5 mg/mL (n = 6, error bars represent standard deviation). ^a–m^ Values are significantly different between the samples (p < 0.001).

Superoxide anion (O_2_ ^•−^) is generated from oxygen (O_2_) by multiple pathways such as oxidation by NADPH oxidase, xanthine or hypoxanthine oxidase. Generally, superoxide anion is converted to hydrogen peroxide by superoxide dismutase (SOD) or reacts with nitric oxide (NO^•^) to form peroxynitrite. Hydrogen peroxide can be further converted to water and oxygen by catalase and glutathione peroxidase. However, superoxide is believed to be the cause of other ROS formations such as hydrogen peroxide, peroxynitrite, and hydroxyl radicals. Superoxide anion is a reduced form of molecular oxygen created by receiving one electron. Superoxide radicals have been observed to kill cells, inactivate enzymes, and degrade DNA, cell membranes and polysaccharides [3,7]. It was, therefore, proposed to measure the comparative interceptive ability of antioxidant extracts to scavenge the superoxide radical [21]. The superoxide radical scavenging effect of the different spices of water extract was compared at the same dose as ascorbic acid (0.5 mg/mL) as shown in Figure 2. When compared to ascorbic acid (36.48%), the superoxide anion radical scavenging activities of marjoram, rosemary, oregano, savory, cumin, basil, thyme, fennel and coriander were higher than that of ascorbic acid. In contrast, the activities of mace, turmeric, clove and caraway were lower than that of ascorbic acid (p < 0.001). We found that marjoram extract had stronger superoxide anion scavenging activity than other spices extract. These results suggest that phenolic compounds in marjoram possess strong antioxidant effects due to superoxide anion radical scavenging in the cellular level.

### 2.4. Hydroxyl Radical Scavenging Activity

**Figure 3:**
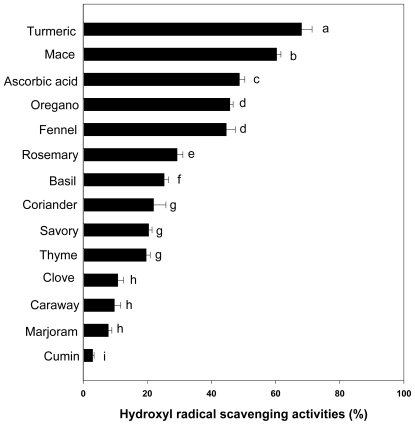
Hydroxyl radical scavenging activities of spices in the concentration of 0.5 mg/mL. (n = 6, error bars represent standard deviation). ^a–l^ Values are significantly different between the samples (p < 0.001).

Among the reactive oxygen species (ROS), hydroxyl radicals are the most reactive and predominant radicals generated endogenously during aerobic metabolism to initiate cell damage *in vivo* [14,22]. We examined the inhibitory action of the samples on deoxyribose degradation which gives an indication of hydroxyl radical scavenging action [23,24]. Hydroxyl radical scavenging activities of selected spices at a concentration of 0.5 mg/mL in relation to ascorbic acid at the same concentration are shown in Figure 3.

The result in decreasing order of hydroxyl radical scavenging activity was turmeric (68.09%) > mace (60.34%) > ascorbic acid (48.72%) > oregano (45.75%), fennel (44.63%) > rosemary (29.29%) > basil (25.25%) > coriander (21.94%), savory (20.43%), thyme (19.64%) > clove (10.78%), caraway (9.72%), marjoram (7.84%) > cumin (2.95%) (p < 0.001). Hydroxyl radical scavenging activity of the turmeric extract was higher than other spices extract (Figure 3). However, hydroxyl radical scavenging activity of turmeric extract showed a different pattern with different methods used such as DPPH and superoxide anion scavenging abilities. Interestingly, the antioxidant activity varies depending on methods used [25,26]. Bioactive compounds in various spices are a complex mixture of compounds. The concentration of the phenolic and flavonoid compounds in various spices varies depending on the cultivar and climate. In addition, Yoo *et al.* [26] reported there are many methods for determination of antioxidant capacity and each method has its own limitation. It was shown that some antioxidant assays give different antioxidant activity trends. Taken together, our results show that the antioxidant activities of various spices have different tendencies, which may depend on the methods used and/or the profile of phenolics.

### 2.5. Correlation Among the Antioxidant Characteristics

Total phenolic and flavonoid contents have been reported to be responsible for the antioxidant activities of botanical extracts. DPPH, hydroxyl radical scavenging activity, and superoxide anion radical scavenging activity have been used to measure antioxidant activity and these results should correlate with those of total phenolic and flavonoids content. A recent report [19,27] demonstrated that some bioactive compounds present in medicinal plants possessed high total antioxidant activity, which was due to the presence of phenolic, carotenoids and flavonoids. A regression analysis was used to correlate the results of the five assays (Table 2).

High correlation coefficients were found between the total phenolic content and DPPH radical scavenging activity (r = 0.9158, p < 0.001). The flavonoid content exhibited moderate correlation coefficients and DPPH radical scavenging activity and superoxide anion radical scavenging activity (respectively, r = 0.5430, r = 0.5598, p < 0.05). However, a non-significant correlation coefficient was found between flavonoid content and total phenolic content (p > 0.05). In addition, no significant correlation coefficient between flavonoid and hydroxyl and superoxide anion radical scavenging activity was found (p > 0.05). As the aluminum chloride method is specific only for flavones and flavonols, total flavonoid content could be underestimated by the method [28], which probably accounts for a lower correlation observed between antioxidant activity and flavonoid count. Liu *et al.* [20] reported a negative correlation between flavonoid content and antioxidant activity.

## 3. Experimental Section

### 3.1. Chemicals and Spices

Ascorbic acid, potassium persulphate, disodium hydrogen phosphate (Na_2_HPO_4_) 2,2-diphenyl-1-picrylhydrazyl (DPPH), deoxyribose, ferric chloride (FeCl_3_), MEDTA, hypoxantine, nitroblue tetrazolium (NBT), xanthine oxidase, Folin–Ciocalteu’s phenol reagent, (+)-catechin, quercetin, sodium nitrite (NaNO_2_), aluminum chloride (AlCl_3_), linoleic acid, thiobarbituric acid (TBA) and trichloroacetic acid (TCA) were purchased from Sigma Co. (St. Louis, MO, USA). Sodium hydroxide (NaOH), hydrogen peroxide (H_2_O_2_) and all solvents used were of analytical grade were purchased from Merck Co. (Darmstadt, Germany). Distilled deionized water (dd. H_2_O) was prepared by Ultrapure TM water purification system (Lotun Co., Ltd., Taipei, Taiwan). The selected spices and herbs were purchased from Taewon Food Industry (Seoul, Korea) in Korea (Table 1).

### 3.2. Extraction Yield of Samples

Spices samples were dried for 48 h to about 4% moisture (dry base) in a hot air-drier at 50 °C. After drying, 4 g of dried sample were extracted with 40 mL of distilled water at a temperature from 80 to 100 °C in reflux for 3 h to give an initial extract (fraction I). The residues were extracted with 60 mL of distilled water at a temperature from 80 to 100 °C for 0.5 h to give fraction II. After cooling to room temperature and then filtering (Whatman No 2), the two fractions were combined and dried under vacuum below 40 °C and weighed to determine the yield. The extracts were completely dried in a freeze-drier and stored at −20 °C until further use.

### 3.3. Measurement of Free Radical Scavenging Activity on DPPH Assay

The free radical scavenging activity of samples (1 mg/mL in DMSO) was measured using the method of Brand-Williams *et al.* [29] with some modification. L-ascorbic acid was used as a positive control. The inhibition percentage was calculated from the following equation: Inhibition % = [(absorbance of control – absorbance of sample)/absorbance of control] × 100. The absorbance was measured by a spectrophotometer (Ultrospec 2100 pro; Amersham Pharmacia Biotech Co., Piscataway, NJ, USA).

### 3.4. Measurement of Superoxide Anion (O_2_ ^•−^) Radical Scavenging Activity

Superoxide radicals were generated by a modified method of Liu *et al.* [30]. The samples (0.5 mg/mL in DMSO) were added to the reaction solution containing 100 μL of 30 mM EDTA (pH 7.4), 10 μL of 30 mM hypoxantine in 50 mM NaOH, and 200 μL of 1.42 mM nitroblue tetrazolium (NBT). After the solution was preincubated at room temperature for 3 min, 100 μL of 0.5 U/mL xanthine oxidase was added to the mixture and the volume was brought up to 3 ml with 50 mM phosphate buffer (pH 7.4). After the solution was incubated at room temperature for 20 min, absorbance was measured at 560 nm. The reaction mixture without xanthine oxidase was used as a blank (A1). The samples (A2) were added to the reaction mixture, in which O_2_ ^•−^ was scavenged, thereby inhibiting the NBT reduction. Absorbance was measured and the decrease in O_2_ ^•−^ was represented by A2–A1. The scavenging activity on superoxide anion radical (SRSA) was calculated by the following equation: SRSA % = (A2 – A1/A1) × 100.

### 3.5. Measurement of Hydroxyl (OH^•^) Radical Scavenging Activity

The scavenging activity of samples in DMSO on the hydroxyl radical (OH^•^) was measured by the deoxyribose method [23] with a slight modification. The deoxyribose assay was performed in 10 mM phosphate buffer (pH 7.4) containing 2.5 mM deoxyribose, 1.5 mM H_2_O_2_, 100 μM FeCl_3_, 104 μM EDTA, and the test sample (0.5 mg/mL). The reaction was started by adding ascorbic acid to a final concentration of 100 μM. The reaction mixture was incubated for 1 h at 37 °C in a water-bath. After incubation, the color was developed by addition of 0.5% thiobarbituric acid followed by ice-cold 2.8% trichloroacetic acid in 25 mM NaOH and heating for 30 min at 80 °C. A control was performed without samples (A1). The sample (A2) was cooled on ice and the absorbance was measured at 532 nm. The hydroxyl radical scavenging activity (HRSA) was calculated by the following equation: HRSA% = (A1 – A2/A1) × 100.

### 3.6. Measurement of Total Phenolic Content Using Folin-Ciocalteu Assay

Total phenolic contents of the extracts were determined spectrophotometrically according to the Folin-Ciocalteu colorimetric method [31]. Because catechin is one of the polyphenol compounds, total phenolic content of hot water extract form 13 spices were expressed as microgram catechin equivalents (CE)/gram.

### 3.7. Measurement of Total Flavonoids

Total flavonoid was determined using the method of Meda *et al.* [28] with minor modifications. In brief, 0.25 mL of sample (1 mg/mL) was added to a tube containing 1 mL of double-distilled water. Next, 0.075 mL of 5% NaNO_2_, 0.075 mL of 10% AlCl_3_ and 0.5 mL of 1 M NaOH were added at 0, 5 and 6 min, sequentially. Finally, the volume of the reacting solution was adjusted to 2.5 mL with double-distilled water. The absorbance of the solution at a wavelength of 410 nm was detected using the Ultrospec 2100 pro spectrophotometers (Section 3.3). Quercetin is a ubiquitous flavonoid, present in many plant extract, was used as standard to quantify the total flavonoid content of hot water extract of the spice extracts. Results were expressed in microgram quercetin equivalents (QE)/gram.

### 3.8. Statistical Analysis

The results were reported as mean ± standard deviation (SD). The significance of differences among treatment means was determined by analysis of variance (one-way ANOVA) using SAS version 8.1 (SAS Institute, Cary, NC, USA). Correlation analyses were performed using the Pearson’s correlation coefficient (r).

## 4. Conclusions

In these results we have focused on hot water extracts obtained from 13 spices commonly used in meat products for their antioxidant properties, which have rarely been reviewed. Results of these antioxidant activities of selected spices using three different assays, such as DPPH assay, superoxide radical scavenging activity, and hydroxyl radical scavenging activity were presented. This analysis technique could provide insight into the variations in the antioxidant profiles between different spices and could help disease prevention and cure using simple herbs and spices. Several spices were found to have high levels of antioxidant capacity and total phenolic compounds. Moreover, the antioxidant capacity, total phenolic content and flavonoid contents of the 13 selected spices were different from each other. Among the selected spices, the clove, thyme and rosemary extracts exhibited higher DPPH radical scavenging activities. In addition, the marjoram, rosemary and oregano extracts exhibited higher superoxide anion radical scavenging activities, and turmeric and mace exhibited higher hydroxyl radical scavenging activities. Interestingly, clove and turmeric showed highest total phenolic content and flavonoid content, respectively, associated with the relatively higher antioxidant activities among these spices. These results suggest that several spices extracts have potential as possible functional ingredients in meat products.

## Figures and Tables

**Table 1:** Extraction yield (%), total phenolic content, and total flavonoids content of how water extract of various spices.

Common name	Botanical Name	Extraction yield (%)	Total phenolic content (μg CE/g)	Total flavonoid content (μg QE/g)
Rosemary	*Rosmarinus officinalis*	19.75 ± 0.43^d^	42.58 ± 1.01^d^	269.84 ± 6.50^b^
Oregano	*Origanum vulgare*	41.33 ± 5.13^a^	23.36 ± 0.93^e^	156.93 ± 9.36^c^
Caraway	*Carum carvi*	12.00 ± 0.72^ef^	9.92 ± 0.11^f^	45.01 ± 2.27^g^
Clove	*Syzygium aromaticum*	19.58 ± 0.14^d^	108.28 ± 7.11^a^	75.97 ± 0.01^f^
Turmeric	*Curcuma longa*	12.50 ± 0.66^ef^	58.28 ± 3.55^b^	324.08 ± 4.34^a^
Thyme	*Thymus vulgaris*	32.03 ± 0.78^b^	7.78 ± 0.31^f^	14.25 ± 0.54^h^
Basil	*Ocimum basilicum*	24.59 ± 0.19^c^	20.25 ± 0.85^e^	131.60 ± 17.83^d^
Marjoram	*Maiorana hortensi*	18.07 ± 0.70^d^	20.44 ± 0.62^e^	157.73 ± 7.06^c^
Mace	*Myristica fragrans*	7.64 ± 0.71^g^	6.50 ± 0.32^f^	7.67 ± 0.83^h^
Fennel	*Foeniculum vulgare*	11.38 ± 0.61^ef^	9.36 ± 0.21^f^	44.76 ± 2.32^g^
Coriander	*Coriandrum sativum*	13.73 ± 1.01^e^	9.22 ± 0.09^f^	3.38 ± 0.09^h^
Savory	*Satureja*	17.67 ± 1.04^d^	48.07 ± 1.61^c^	35.19 ± 5.84^g^
Cumin	*Cuminum cyminum*	10.27 ± 0.79^fg^	10.17 ± 0.68^f^	101.34 ± 4.08^e^

Note: The spices samples were refluxed with hot water at a temperature from 80 to 100 °C for 3 h, and the extraction was repeated two times. Total phenolic content expressed as milligrams of catechin equivalent (CE)/g of extract; Total flavonoid content expressed as milligrams of quercetin quivalent (QE)/g of extract; The extraction yield (%) was calculated as (g of extract/g of dried spices) × 100.

a–h Values are means ± S.D. significant difference between the samples (n = 6) (p < 0.05).

**Table 2:** Correlations (r^a^) between different antioxidant capacity parameters (by DPPH, hydroxyl, and superoxide radical scavenging activity) and total phenolic contents or flavonoid contents of various spices’ water extracts.

	TPC^b^	Flavonoid	DPPH^c^	HRSA^d^	SRSA^e^
TPC		0.3164	0.9158^***^	−0.1422	−0.1052
Flavonoid			0.5430^*^	−0.2599	0.5598^*^
DPPH				−0.4218	0.0434
HRSA					−0.1422

a r, correlation coefficient;

b TPC, total phenolic content;

c DPPH radical scavenging activity;

d HRSA, hydroxyl radical scavenging activity;

e SRSA, superoxide anion radical scavenging activity.

Significance level at ^**^p < 0.01 and ^***^p < 0.001.
